# Retraction: Chemopreventive efficacy of *Andrographis paniculata* on Azoxymethane-induced aberrant colon crypt foci *In Vivo*

**DOI:** 10.1371/journal.pone.0328989

**Published:** 2025-07-28

**Authors:** The PLOS One Editors

After the publication and correction of this article [1,2], concerns were raised regarding results presented in Figs 4–6, and Tables 1–3 and 5.

Specifically,

- The Fig 4E panel in [1] and the Fig S1C panel in [3, retracted in 4] appear to partially overlap
- The following panels appear similar:
  - Fig 5A in [1] and Fig 3a panel A in [5]
  - Fig 5B in [1] and Fig 1B in [3, retracted in 4]
  - Fig 5C in [1] and Fig 5A in [6]
  - Fig 6E in [1] and Fig 5a panel D in [5] when rotated 180°
- There appear to be overlaps in some of the values in the following tables. It is unclear whether these results represent data obtained from the same or different conditions.
  - Table 1 in [1] and Table 2 in [5]
  - Table 2 in [1] and Table 3 in [5]
  - Table 3 in [1] and Table 4 in [5]
  - Table 5 in [1] and Table 1 in [5]

The corresponding author stated that they were unable to retrieve the original data underlying the results of this study. In light of the above concerns that question the reliability of the reported results and conclusions, the *PLOS One* Editors retract this article.

NAH, RPYY, SI, WN, SAMK, and HES either did not respond directly or could not be reached. MAA did not respond to the final editorial decision.
